# Mechanism of tacrolimus in the treatment of lupus nephritis

**DOI:** 10.3389/fphar.2024.1331800

**Published:** 2024-05-07

**Authors:** Ming Wang, Jing Zhou, Qiyan Niu, Hongyue Wang

**Affiliations:** Department of Nephrology, The First Hospital of Jilin University, Changchun, China

**Keywords:** tacrolimus, calcineurin inhibitor, lupus nephritis, systemic lupus erythematosus, regulatory mechanisms

## Abstract

Systemic lupus erythematosus (SLE) is a complex autoimmune disorder, with more than half of the patients developing lupus nephritis (LN), which significantly contributes to chronic kidney disease (CKD) and end-stage renal disease (ESRD). The treatment of lupus nephritis has always been challenging. Tacrolimus (TAC), an effective immunosuppressant, has been increasingly used in the treatment of LN in recent years. This review aims to explore the mechanisms of action of tacrolimus in treating LN. Firstly, we briefly introduce the pharmacological properties of tacrolimus, including its role as a calcineurin (CaN) inhibitor, exerting immunosuppressive effects by inhibiting T cell activation and cytokine production. Subsequently, we focus on various other immunomodulatory mechanisms of tacrolimus in LN therapy, including its effects on T cells, B cells, and immune cells in kidney. Particularly, we emphasize tacrolimus’ regulatory effect on inflammatory mediators and its importance in modulating the Th1/Th2 and Th17/Treg balance. Additionally, we review its effects on actin cytoskeleton, angiotensin II (Ang II)-specific vascular contraction, and P-glycoprotein activity, summarizing its impacts on non-immune mechanisms. Finally, we summarize the efficacy and safety of tacrolimus in clinical studies and trials. Although some studies have shown significant efficacy of tacrolimus in treating LN, its safety remains a challenge. We outline the potential adverse reactions of long-term tacrolimus use and provide suggestions on effectively monitoring and managing these adverse reactions in clinical practice. In general, tacrolimus, as a novel immunosuppressant, holds promising prospects for treating LN. Of course, further research is needed to better understand its therapeutic mechanisms and ensure its safety and efficacy in clinical practice.

## 1 Introduction

SLE is an autoimmune disorder intricately linked to genetic predisposition, environmental triggers, hormonal imbalances, immune dysregulation, and the breakdown of self-tolerance towards autoantigens. It predominantly afflicts women of childbearing age, with approximately 50% of affected individuals presenting definitive evidence of renal impairment. LN stands as one of the most severe manifestations of SLE. An estimated 10% of LN patients will inevitably advance to ESRD (Almaani et al., 2017). LN currently remains incurable, but appropriate early clinical intervention can preserve residual renal function and achieve long-term remission. Conversely, in the absence of timely and standardized therapeutic interventions, renal function typically undergoes progressive deterioration, culminating in recurrent episodes and a markedly heightened mortality rate. The precise pathogenesis leading to LN is currently uncertain, but it is unequivocally established that aberrations in cell death and cellular debris clearance, augmented nucleic acid recognition and interferon signaling, genetic anomalies, lymphocytic dysregulation, aberrant complement system activation, and perturbed cytokine milieu collectively contribute to the initiation and progression of both SLE and LN (Lichtnekert et al., 2022). It has been suggested that augmented programmed cell death and impaired neutrophil-specific clearance of apoptotic remnants, resulting in the breakdown of immune tolerance to nuclear autoantigens, may constitute the incipient trigger for systemic autoimmunity in SLE (Yang et al., 2019). Simultaneously, these events engender heightened availability of pattern recognition receptor ligands and opsonized antigens, thereby augmenting innate immune responses and phagocytic activity, precipitating the initiation of IFN-a-mediated antiviral defense mechanisms that assail healthy cells and tissues across the systemic milieu, including the kidneys (Munroe and James, 2015). Significantly, T cells and B cells constitute pivotal components of the immune repertoire in both SLE and LN. Diminished cytotoxicity of CD8 T cells confers an elevated susceptibility to infections (Gravano and Hoyer, 2013). A variety of CD4^+^ T helper cell (Th cell) subsets, encompassing aberrant activation of Th1, Th2, Th17 lineages along with their respective cytokines, alongside regulatory T cell (Tregs) dysfunction, are intricately linked to the immune-mediated pathogenesis of both SLE and LN (Suárez-Fueyo et al., 2016; Koga et al., 2017; Li et al., 2022).

**FIGURE 1:**
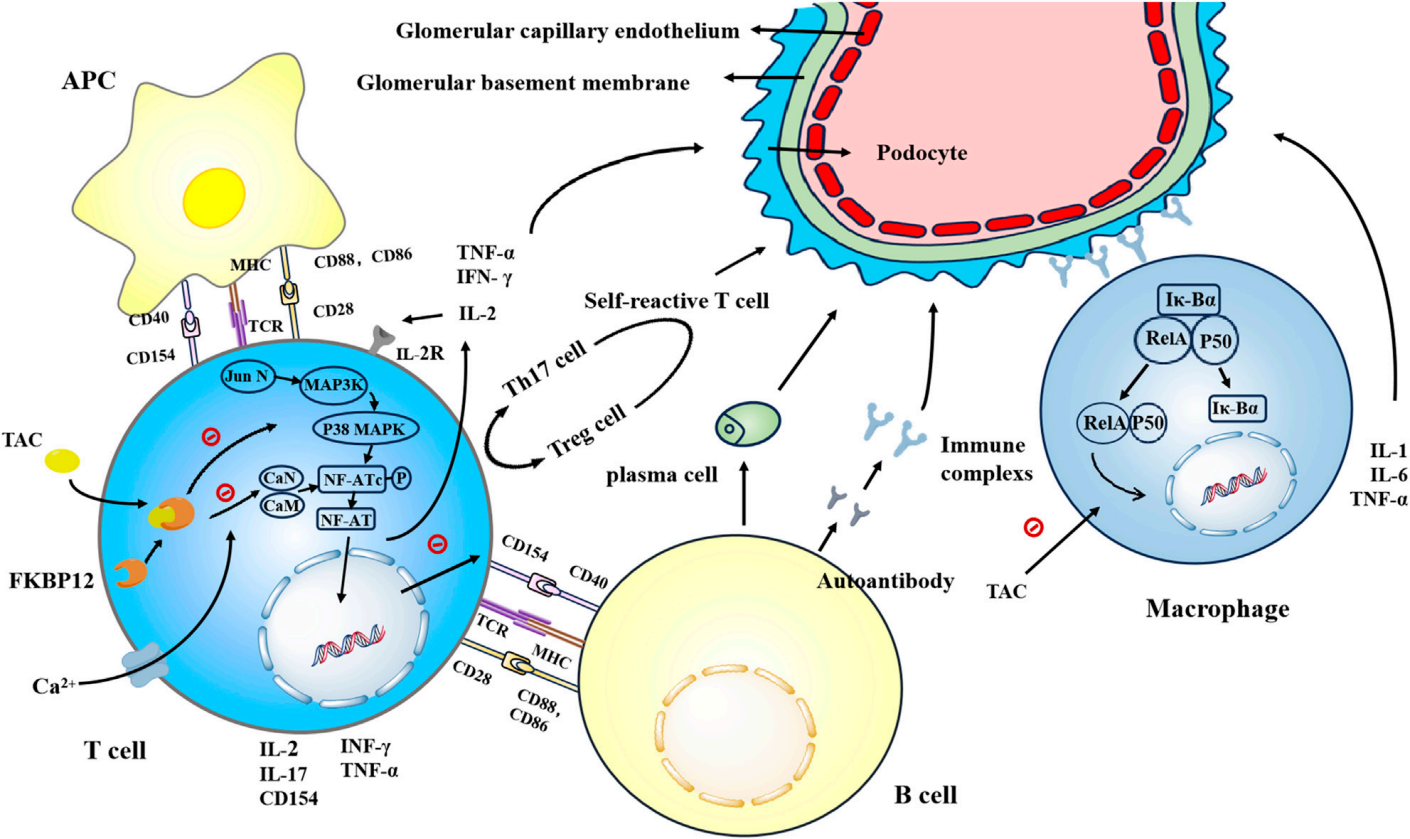
Tacrolimus enhances the affinity of FKBP12 for CaN by binding to FKBP12, thereby inhibiting the dephosphorylation and translocation of NFATc in a concentration-dependent manner. This inhibition suppresses the generation of key cytokines such as IL-2, TNF-α, and IFN-γ, as well as IL-17 and CD154. Tacrolimus inhibits the binding of CD154 to CD40, thereby suppressing the proliferation and differentiation of B cells. Additionally, tacrolimus inhibits the generation of autoreactive T cells. Tacrolimus inhibits the activation of the JNK and p38 signaling pathways triggered by antigen recognition, thereby suppressing T cell activation. Tacrolimus prevents the nuclear translocation of NF-κB subunits RelA, p50, c-Rel, and others, inhibiting DNA binding and reducing the generation of IL-1, IL-6, and TNF-α.

Moreover, defective B cell tolerance and perturbed B cell-T cell interactions mayprecipitate BERRANT B cell maturation and autoantibody production (Yap and Chan, 2019). Within the renal milieu, the TNF superfamily orchestrates renal injury via instigation of inflammation, cellular apoptosis, and extracellular matrix deposition, thereby impeding glomerular filtration function (Sanchez-Niño et al., 2010). These insights into LN pathogenesis furnish a theoretical framework underpinning the plausibility of specific targeted therapeutic modalities. Mitigation of CKD and forestalling the onset of ESRD constitutes the primary imperative in the management of LN. Given the heightened propensity for adverse renal outcomes in proliferative LN, there has been a discernible shift in focus towards therapeutic interventions targeting classes III and IV LN in clinical practice. During the active phase of LN, induction therapy typically spans a duration of 3–6 months. The crux of therapeutic intervention lies in effecting either complete or partial amelioration of the clinical manifestations of renal injury. Along with anti-inflammatory treatment, potent immunosuppressants were given to interrupt the autoimmune pathway and induce disease quiescence. Subsequently, a protracted phase of maintenance therapy ensues, with the primary objective being dose optimization of immunosuppressive agents, attenuation of their adverse effects, ensuring therapeutic stability, and precluding further renal unit depletion precipitated by renal flares. At present, the commonly used immunosuppressive drugs include Mycophenolate mofetil (MMF) and cyclophosphamide (CYC). Nevertheless, the concern over the toxicity profile of CYC is increasingly becoming palpable. MMF, notably, has been implicated in augmenting the risk of ESRD in LN patients, especially those afflicted with diffuse proliferative LN. Derived from the fungus *Streptomyces* tsukubaensis, tacrolimus was first isolated in 1984 in the vicinity of Mount Tsukuba, Ibaraki Prefecture, Japan. In recent years, it has gradually garnered the attention of clinicians. This is a calcineurin inhibitor (CNI), which is a lipophilic macrolide with properties similar to cyclosporine. In the management of LN, tacrolimus has demonstrated superiority over CYC not only in terms of efficacy for induction therapy over a 6-month timeframe but also in its markedly superior safety profile (Zhang et al., 2016; Lee and Song, 2022). Moreover, it is as effective as MMF against proliferative LN (Mok et al., 2016). In this article, we initially elucidate the currently acknowledged therapeutic mechanisms of tacrolimus in LN encompassing both immune and non-immune perspective Figure 1. Furthermore, we delve deeper into the assimilation of contemporary findings and potential molecular underpinnings to furnish novel insights for the management of LN.

## 2 Immune mechanisms

### 2.1 Tacrolimus inhibits the activity of calcineurin (CaN)

Calcineurin, a calcium/calmodulin (CaM)-dependent protein phosphatase, consist of a calmodulin-binding catalytic subunit (calcineurin a, CN-A) and a calmodulin-binding regulatory subunit (calcineurin b, CN-B) (Rusnak and Mertz, 2000). Its activation requires the release of intracellular Ca^2+^ induced by co-stimulation of the T cell receptor (TCR) and antigen-presenting cells (APC). During this process, Ca^2+^ binds to CN-B, thereby facilitating the activation of CaN through the interaction between Ca^2+^-calmodulin and CN-A (Stemmer and Klee, 1994). With the assistance of activated calcineurin, the nuclear factor of activated T cells (NF-ATc) are phosphorylated and translocated into the nucleus to bind to protein dimers induced by the T cell receptor (NF-ATn) signaling pathway to form NFAT(Hogan et al., 2003), which subsequently trigger transcriptional and translational upregulation of numerous cytokines, including Interleukin-2 (IL-2), Tumor Necrosis Factor-alpha (TNF-α), and Interferon-gamma (IFN-γ). IL-2 has been shown to be a crucial growth factor that promotes the proliferation of effector and memory cells. The binding of IL-2 to its receptor can activate a kinase cascade, which ultimately promotes the proliferation and differentiation of T cells. Upon receiving these signals, activated T lymphocytes synthesize nucleotides and then proliferate and differentiate into multiple T cell subsets (Issa et al., 2013). These cells release multiple inflammatory mediators upon direct contact with renal tubular endothelial and epithelial cells, mediating renal injury.

Tacrolimus, a naturally occurring CaN, functions as a lipophilic prodrug. It enters cells by binding to FK506-binding protein 12 (FKBP12), which enhances FKBP’s affinity for CaN(Shaw et al., 1995). Tacrolimus effectively inhibits the dephosphorylation and translocation of NFATc in a concentration-dependent manner, thereby suppressing cytokine production in memory CD4 T cells, particularly the IL-2 gene. It is noteworthy that the inhibitory effect of tacrolimus on IL-2 expression is also modulated by patient genotypes. For instance, patients with the IL-2-330GG genotype exhibited markedly elevated blood concentrations when treated with tacrolimus, hence requiring lower doses of tacrolimus (Y et al., 2015).

### 2.2 The regulatory effect of tacrolimus on cytokines

Cytokines play a pivotal role in the pathogenesis and progression of SLE and LN. Studies have demonstrated that activated T cells participate in the systemic inflammatory response by increasing the release of cytokines and pro-inflammatory factors such as interleukin-1 (IL-1), interleukin-6 (IL- 6), and TNF-α(C A, 2000, John W and Larry, 2006). Cytokines also play a crucial role in the development and activation of adaptive immunity. They enhance Th1, Th2, and Th17 pathways, as well as follicular helper T cell (Tfh) mechanisms, while simultaneously attenuating Treg mechanisms, leading to T cell activation and immune dysregulation (Melissa and Judith, 2015). For example, IL-2 stimulates T cell activation and promotes their differentiation into Th1 or Th2 cells (C A, 2000), with Th1 cells characterized by the production of IL-2 and IFN-γ, while Th2 cells produce IL-4, IL- 5, and IL-13 (A et al., 1999, T R et al., 1986). Th17 cells, defined by their production of IL-17, also act as inducers of inflammation (Itay et al., 2014).

In SLE, IFN-α is the first cytokine found to be elevated, and its levels correlate with disease activity (S R and TJ, 1982). It promotes the proliferation and differentiation of monocytes into antigen-presenting cells and B cells into plasma cells. TNF-α is primarily produced through the interaction between monocytes and activated T cells, with a minor portion synthesized by intrinsic renal cells. Research has shown that anti-double-stranded DNA (anti-dsDNA) antibodies can stimulate mesangial cells and proximal renal tubular epithelial cells to secrete TNF-α, thereby promoting the production of IL-1β and IL-6 by these cells during the early phases of SLE (Susan et al., 2005). Furthermore, inflammatory factors such as IL-10 and hyaluronic acid (HA) also participate in renal damage, playing significant pro-inflammatory roles in LN (Amy M et al., 2002).

Tacrolimus, as an effective cytokine inhibitor, has received widespread attention for its role in alleviating systemic inflammatory responses. Studies have shown that tacrolimus, similar to cyclosporine A, exhibits comparable effects in inhibiting cytokines, particularly in suppressing Th1 cytokines (such as IL-2 and IFN-γ) and Th17 cytokines (such as IL-17) (Fredrik et al., 2017). Additionally, tacrolimus significantly inhibits cytokines regulated by NFAT, such as IL-2, IFN-γ, and colony-stimulating factor 2 (CSF2) in post-kidney transplant patients. Following tacrolimus treatment, the expression of these cytokines gene is downregulated to varying degrees. For example, when the concentration of tacrolimus exceeded 15 μg/L after 1.5 h, the inhibitory effect on cytokines was potent, with residual gene expression (RGE) not surpassing 10%. Lower concentrations of tacrolimus may lead to more variable responses, with RGE ranging from 2.5% to 68.7% (Sara et al., 2017). *In vitro* studies confirmed that tacrolimus could inhibit the production of TNF-α and IL-1β induced by anti-CD3/CD28 at concentrations less than 1 ng/mL (S et al., 2000). It has been suggested that this process may involve the interaction between T cells and monocytes, such as the attachment of CD40 and CD40 ligand, or the interaction of CD2 and lymphocyte function antigen-3 (LFA-3), rather than being primarily caused by the blockade of T cell-produced cytokines such as IL-2.

Nuclear factor-kappaB (NF-κB) is a transcription factor that plays a crucial role in inflammation and renal tubulointerstitial injury (Qiutang and Inder M, 2002). Its downstream inflammatory mediators such as IL-1, IL-6, and GM-CSF are also believed to be associated with histopathological indicators of renal tubulointerstitial and/or renal function (Ling et al., 2008). NF-κB is an important heterodimer formed by p50 and p56 subunits. In the resting state, NF-κB remains inactive by binding with its endogenous inhibitor κB-α (IκB-α), thus preventing the translocation of p65 into the nucleus. However, the IκB kinase (IKK) complex is activated and phosphorylated under certain stimuli. Subsequently, IκB-α protein is degraded, leading to the dissociation of NF-κB from IκBα and the release of p65 heterodimers, thereby regulating the transcription of various genes (S et al., 1998). Early studies have confirmed that CNIs affect the expression of inflammatory mediators through NF- κB-related pathways. Frantz et al. proposed that CaN can promote the secondary transcription of IL- two through NF-κB.

The action of CNIs and CaN can promote the binding of NF-κB to IκB, thereby inhibiting the production of NF-κB-mediated pro-inflammatory factors (B et al., 1994). Recent studies suggest that specific drugs such as tacrolimus and IκBα super-repressors block the entry of NF-κB subunits RelA, p50, c-Rel, and others into the nucleus. Finally, inhibition of DNA binding reduces the generation of inflammatory factors IL-1, IL-6, and TNF-α (Ting et al., 2017).

### 2.3 Tacrolimus suppresses T-cell activation by blocking antigen recognition-triggered JNK and p38 MAPK signaling pathways

The signaling pathways of T cell activation are complex and variable. Besides inflammation mediators directly inducing T cell activation, the p38 MAPK signaling pathway also plays a crucial role in T cell development and activation (Ryan et al., 2007). This pathway remains dormant in the absence of stimulation, but upon encountering stimuli such as growth factors, cytokines, radiation, or changes in osmotic pressure, MAP3K gradually undergoes phosphorylation. Activated P38MAPK acts as an upstream activating factor for NFAT, influencing the activation of NFATc, and also affects the transcription of cytokines such as IL-2, IFN- γ, and TNF- α, thereby directly activating T cells (Chia-Cheng et al., 2003). Tacrolimus effectively inhibits this pathway, as it can inhibit the activation of JNK and p38 pathways in a non-CaN-dependent manner (S et al., 2001). Research by Vafadari et al. showed that tacrolimus inhibited p38MAPK phosphorylation in renal transplant patients in a dose-dependent way, with an inhibitory efficiency of more than 60% in T cells (Ramin et al., 2012). Similarly, experiments by Miroux et al. also confirmed that tacrolimus can significantly inhibit T cell proliferation, particularly showing significant inhibition of Tregs proliferation at high doses. For instance, the inhibition rate of Treg proliferation was about 35% at tacrolimus concentrations up to 40 ng/mL, while it could reach 70% at 400 ng/mL, and this inhibitory effect was independent of the CaN/NFAT pathway (Céline et al., 2012). However, the inhibitory effect of tacrolimus on monocytes is relatively limited. This inhibitory effect was found to be no more than 35% in monocytes, suggesting that it may have a more limited inhibitory effect on intrinsic immune cells (Nynke M et al., 2017).

### 2.4 Tacrolimus inhibits the proliferation and differentiation of B cells

The breakdown of central and peripheral tolerance mechanisms that prevent the development and survival of self-antigen-reactive B cells may cause an increase in self-reactive B cells, leading to SLE and LN (Karrar and Cunninghame Graham, 2018). T cells largely influence the effect of tacrolimus on B cells. The production of antibodies relies on a specialized subpopulation of CD4^+^ T cells known as Tfh cells, which play an essential role in mediating B cell-mediated humoral immunity. Tfh cells promote the generation and proliferation of B cells into memory B cells, as well as Ig-type transition, maturation, and differentiation through germinal center (GC) reaction and extrafollicular antibody responses (Song and Craft, 2019). According to a study, the number of circulating Tfh cells is elevated in patients with SLE and appears to be correlated with autoantibody levels and/or disease severity (Simpson et al., 2010). Tacrolimus has been shown to dampen humoral immunity by suppressing Tfh cells. Experiments by Kraaijeveld R et al. discovered that tacrolimus significantly inhibited the proliferation and development of Tfh-like cells, preventing them from efficiently activating B cells, thereby reducing autoantibody secretion and immune complex formation (Kraaijeveld et al., 2019). Firstly, IL-21 is secreted by Tfh cells in response to stimulation and plays a crucial role in B-cell proliferation, activation, and plasma cell generation (Baan et al., 2014). The level of IL-21 expression was lower after applying tacrolimus. This suggests that the impaired assistance of T-cell-dependent B cells may be related to reduced levels of IL-21 produced by tfh-like cells (Kraaijeveld et al., 2019). Secondly, T cell activation requires the binding of co-stimulatory molecules on T cells to their respective ligands on APC. The interaction between CD40L and CD40, members of TNF family, was proven to be vital for B-cell development, antibody and IL-12 production, germinal center formation, optimal T-cell-dependent antibody responses, and CD8 T-cell efficient function (Karrar and Cunninghame Graham, 2018). Moreover, it has been well established that CD40L overexpression on CD4 T cells is involved in the pathogenesis of SLE (Desai-Mehta et al., 1996; Koshy et al., 1996). Tacrolimus can completely block the translocation of NF-AT cytoplasmic components, thereby downregulating the expression of the CD40L gene and inhibitingthe development of B cells and the production of autoantibody. Interestingly, Elizabeth F. Wallin et al. observed that Tfh cells are more likely to be affected by tacrolimus than other subsets of CD4 T cells, which may be related to the different expression levels of NF-AT in the cells (Vaeth et al., 2014; Gustavo J et al., 2016).

An *in vitro* experiment demonstrated tacrolimus could impact humoral immunity by directly inhibiting the proliferation of naive B cells, plasmablast differentiation, and immunoglobulin expression. However, there is no study that finds tacrolimus to have this effect on total B cells (R et al., 2015). Similarly, Wallin EF et al. observed in their experiments that tacrolimus prevented B cell development and antibody generation. However there were no apparent differences in the absolute number of circulating B cells or the proportion of LN B cells between tacrolimus-untreated and tacrolimus-treated patients (Elizabeth F et al., 2018). In summary, there are limited studies on the direct effects of tacrolimus on B cells. Therefore, its specific mechanism needs further elucidated.

### 2.5 Toll-like receptor signaling pathway

Toll-like receptors (TLRs), crucial protein molecules in nonspecific immunity associated with systemic autoimmunity, are implicated in the initiation of SLE and LN (Georg and Hans-Joachim, 2015). For instance, TLR9 and TLR7 exhibit immunostimulatory properties capable of activating B cells and APCs. Furthermore, the exact pathogenic mechanism remains elusive (Beverly D et al., 2014). A study provided evidence of tacrolimus inhibiting TLR signaling in liver transplant patients (Jessica et al., 2013). Moreover, a recent study on sepsis-associated acute kidney injury found that medium-high doses of tacrolimus significantly inhibit TLR4-related signaling pathways, thus reducing the inflammatory response and cellular injury (Xueqing et al., 2021). In conclusion, the suppressive effect of tacrolimus on Toll-like receptor signals may be one of the mechanisms for the treatment of SLE and LN. Its specific mechanism needs to be discovered by further studies.

## 3 Non-immune mechanism

### 3.1 Tacrolimus stabilizes the actin cytoskeleton of podocytes and inhibits podocyte apoptosis

Podocytes are specialized structures covering the outer surface of the glomerular basement membrane (GBM), comprising structural proteins like actin and myosin. These proteins regulate glomerular filtration through dynamic contraction and relaxation (Pavenstädt et al., 2003). Podocyte injury is a common feature of several renal diseases, including LN, resulting in rearrangement of the actin cytoskeleton and loss of podocytes. Followed by the development of proteinuria. A study suggested that the anti-albuminuric and nephroprotective effects of tacrolimus may be attributed in part to the stabilization of the actin cytoskeleton and the maintenance of podocyte numbers (Liao et al., 2015). This mechanism is elaborated upon in the study by Peleg Y et al. (Peleg et al., 2020). Phosphorylated synaptopodin (Synaptopodin-P) binds to 14-3-3 and stabilizes the actin cytoskeleton of podocytes. Under the action of CaN, Synaptopodin-P is dephosphorylated to Synaptopodin, which can be disrupted by cathepsin L, leading to synaptic peptide disruption and cytoskeletal instability. Tacrolimus suppresses these processes by inhibiting Synaptopodin-P dephosphorylation, thereby reducing proteinuria in LN patients. In addition, tacrolimus can stabilize the cytoskeleton by downregulating TGF-β1. TGF-β, along with certain immune complexes found in SLE and LN patients, can cause podocyte damage (Zhao et al., 2005; Herman- Edelstein et al., 2013). In mouse podocytes, TGF-β1 enhances damage to the actin cytoskeleton, and tacrolimus inhibits the production of TGF-β1 to stabilize it. Likewise, Liao R, et al. observed that TGF-β1 may damage the actin cytoskeleton, a condition that can be restored and stabilized through tacrolimus treatment (Liao et al., 2015). An animal study additionally discovered that tacrolimus reduced podocyte apoptosis induced by TGF-β1 and prevented podocyte fusion, thus potentially reducing proteinuria in LN and preserving renal function. A large foot-process width usually indicates more severe podocyte fusion, which is involved in urinary protein production. The width of the foot processes was significantly reduced following tacrolimus administration (Shen et al., 2016). Furthermore, this experiment revealed that tacrolimus inhibits mitochondria-dependent podocyte apoptosis by suppressing the MAPK signaling pathway, a mechanism consistent with the one mentioned earlier.

### 3.2 Tacrolimus restored FKBP12 expression in F-actin cytoskeleton and mitigated the exacerbation of injured podocyte protrusion formation

FKBP12 has been proven to be a protein that binds to tacrolimus. Recent studies have shown that FKBP12 maintains actin fibers and tacrolimus protects podocytes through a mechanism independent of calcineurin (Yasuda et al., 2021). In normal podocytes, FKBP12 is distributed along the actin cytoskeleton and binds to F-actin. It maintains F-actin integrity by interacting with 14-3-3 and synaptopodin. In the podocyte injury model, the expression of FKBP12 was significantly decreased, resulting in decreased expression of 14-3-3 and disruption of the 14-3-3-synaptopodin connection. Tacrolimus treatment restores FKBP12 expression in the F-actin cytoskeleton and ameliorates podocyte damage. The precise mechanism by which tacrolimus strengthens the interaction between 14-3-3-synaptopodin-P and FKBP12-synaptopodin-P is unclear, but it may involve tacrolimus affecting the isomerase activity of FKBP12. Interestingly, it is known that CsA and tacrolimus are both calcium phosphatase inhibitors with distinct structural formulas. The study also found that, unlike FKBP, cyclophilin A (CyPA) was expressed in some mesangial cells as well as podocytes. This variation in distribution may indicate that tacrolimus has a smore pronounced selective impact on podocytes compared to CsA, which could be attributed to the differing pharmacological actions of tacrolimus and CsA.

### 3.3 Tacrolimus induces Ang II-specific vasoconstriction to decrease GFR, thereby reducing proteinuria

Tacrolimus-induced constriction of afferent arterioles is mediated by the activation of the RhoA (Ras homolog family member A)/ROCK (Rho-associated protein kinase)/MYPT-1 (myosin phosphatase targeting subunit 1) pathway. The RhoA/ROCK pathway is implicated in the pathogenesis of various kidney diseases, including diabetic kidney disease, acute kidney injury (Komers, 2013; Wang et al., 2018), and LN-associated kidney injury. Fengyuan Tian et al. demonstrated that Fasudil, a Rho family small GTP-binding protein (Rho GTPase) inhibitor, can reduce podocyte damage in LN by interfering with upstream CaMK4/Rho GTPase signaling pathway in podocytes in lupus-prone mice (Tian et al., 2023). A recent study demonstrated that tacrolimus can cause Ang II-induced vasoconstriction through the activation of the RhoA/ROCK pathway (Wang et al., 2022). In mammals, there are 20 members of the Rho GTPases (Shimokawa et al., 2016), among with RhoA acts as a molecular switch capable of alternating between GTP-bound and GDP-bound forms. RhoA binds to GDP and subsequently converts to GTP-bound RhoA, which becomes activated upon translocation to the membrane. Its downstream effector, ROCK, phosphorylates MYPT-1, thereby regulating vasoconstriction. Activation of this pathway enhances the resistance of afferent small arteries, reduces the GFR, and alleviates proteinuria. Tacrolimus may enhance Ang II-specific vasoconstriction, potentially attributed to its augmentation of reactive oxygen species (ROS) production, which may enhance the activity of the RhoA/ROCK/MYPT-1 pathway. Furthermore, the presence of tacrolimus substantially enhances intracellular Ca2+ release triggered by Ang II. This effect may stem from the complex formed by tacrolimus binding to its receptor FKBP, which potentially displaces FKBP from IP3R or ryanodine receptors, thereby enhancing the opening probability of IP3R and ryanodine receptor channels (Wang et al., 2022). Further research is needed to elucidate the exact mechanism.

### 3.4 Tacrolimus inhibits P-glycoprotein (P-gp) activity through a dual mode of action, overcoming P- gp-related drug resistance

P-glycoprotein (P-gp) is widely distributed in normal human tissues and acts as an efflux pump for toxins and drugs, including glucocorticoids (GCs) and immunosuppressive agents (Ahmed Juvale et al., 2022). Several studies have shown that excessive or hyperfunction of P-gp on lymphocytes may lead to the expulsion of glucocorticoids from the cell, impairing regular intracellular hormone activity. This phenomenon has been associated with the development of glucocorticoid resistance in patients with SLE and LN (Tsujimura et al., 2005a; Tsujimura et al., 2005b). Recent research suggests that tacrolimus could offer a safer and superior therapeutic approach for managing patients with refractory and recurrent LN. A portion of this benefit is attributed to the alleviation of P-gp-mediated glucocorticoid resistance (Edavalath et al., 2022). Tacrolimus acts as a P-gp inhibitor with a dual mode of action to reduce P-gp activity. It has been previously suggested that IL-2 upregulates P-gp expression by activating the transcription factor YB-1, leading to a significant reduction in intracellular glucocorticoid concentration *in vitro* (Tsujimura et al., 2004; Kansal et al., 2016).

Tacrolimus can inhibit IL-2 synthesis by interfering with the NF-AT pathway, thereby downregulating P-gp expression. Consequently, by inhibiting the export of glucocorticoids from the cell, tacrolimus can effectively increase the intracellular concentration of GCs, thereby alleviating drug resistance during the treatment of SLE and LN. Interestingly, a study demonstrating that tacrolimus overcomes P-gp-mediated treatment unresponsiveness in refractory rheumatoid arthritis (RA) patients found that the primary mechanism of P-gp inhibition by tacrolimus is through functionally competitive drug rejection rather than a reduction in P-gp molecules on cells (Suzuki et al., 2010).

### 3.5 Tacrolimus attenuates GC resistance through some inhibitory signaling pathways and decreases Thl7 expression

Approximately 30% of individuals suffering from autoimmune diseases exhibit inadequate response to GC therapy (Seki et al., 1998; Chikanza and Kozaci, 2004). Research has established that IL-17 and TNF-α impede GC responsiveness in inflammatory bowel disease (Fujino et al., 2003; Bantel and Schulze-Osthoff, 2013). GC resistance is also prevalent among patients with SLE and LN. Besides the previously mentioned P-gp overexpression, the mechanism of GC resistance may also involve certain signaling pathways and Th17 cells. B. Bouazza et al. found that p38-MAPK phosphorylation-mediated reduction in glucocorticoid receptor nuclear translocation is another potential mechanism contributing to GC resistance (Bouazza et al., 2014). Y. Guan et al. discovered that macrophage migration inhibitory factor stimulates T cells and macrophages to produce more pro-inflammatory factors via MAPK signaling, exacerbating inflammation and contributing to glucocorticoid resistance in SLE patients (Guan et al., 2015). Tacrolimus alleviates glucocorticoid resistance by inhibiting the activity of the JNK and p38 pathways independently of CaN. Additionally, Toll-like receptors may potentially be linked to GC sensitivity in SLE patients, according to certain research (Kawai and Akira, 2010). However, further investigation is necessary to ascertain the precise mechanism and whether tacrolimus attenuates GC resistance through this pathway. Thl7 cells are closely linked to GC resistance. Treatment of Th17 cells with CsA significantly suppressed the expression of several human genes, including IL-17A and IL-17F. Tacrolimus elicited a similar inhibitory effect (Schewitz- Bowers et al., 2015). The result was consistent with their clinical efficacy in treating GC resistance, suggesting that TAC may selectively inhibit Thl7 cells to alleviate GC resistance. Moreover, specific cytokines, such as TNF-α and IFN-γ, markedly attenuate GR signaling by upregulating the expression of GRβ. Tacrolimus suppresses the expression of these cytokines while enhancing GR signaling (Barnes, 2013). Additionally, tacrolimus may modify the hormone-binding capacity of GR, potentially by modulating the TPR protein component within the receptor complexes (Davies et al., 2005).

## 4 Adverse effects of long-term use of tacrolimus

The major adverse effects of tacrolimus include nephrotoxicity, neurotoxicity, diabetes, gastrointestinal disorders, hypertension, infections, and malignant complications, and these tend to be more frequent or even aggravation of the severe at higher concentrations (Suarez-Kurtz and Struchiner, 2024). Long-term use of tacrolimus may lesd to nephrotoxicity or ven aggravation of pre-existing nephritis. In previous studies, most tacrolimus-related nephrotoxic events were reported in kidney and liver transplant recipients, and LN patients may be different from these patients. A recent prospective study with a median follow-up of 60 months indicated relatively low rates of kidney recurrence with tacrolimus maintenance therapy for active LN Approximately 21.3% of patients experienced renal relapse with an average dose of 2 mg/d (Zhang et al., 2022). However, it’s plausible that the lower maintenance dose of tacrolimus during this phase may have less impact on renal function deterioration. Tacrolimus may lead to hyperglycemia by affecting insulin secretion and altering tissue sensitivity to insulin. An earlier study found a 4% incidence of new-onset type 1 diabetes in kidney transplant recipients receiving triple therapy (tacrolimus, corticosteroids, and azathioprine), while patients on dual therapy (tacrolimus and corticosteroids) had a 5.6% incidence after a 3-month follow-up (Neuhaus et al., 1997). However, Zhang et al. found similar rates of hyperglycemia in tacrolimus-treated LN patients compared to those treated with mycophenolate mofetil or azathioprine. Tacrolimus-related neurotoxicity is most commonly observed after intravenous administration (Kelly et al., 1995). Neurological events post-tacrolimus treatment are also predominantly studied in organ transplant recipients. Mild neurological events include insomnia, mild tremors, headaches, photophobia, nyctalopia, and hypervinylosis. In major clinical trials, tremors occurred in 35%–56% of tacrolimus recipients, headaches in 20%–64%, insomnia in 24%–32%, and sensory disturbances in 14%–40%(1994a, 1994b, A D et al., 1997). Severe neurotoxic effects, such as seizures, expressive aphasia, coma, and delirium, are rare and associated with higher tacrolimus blood concentrations(E et al., 2007). In a large prospective study, over 50% of patients experienced major neurological adverse events related to elevated trough plasma concentrations(E et al., 2007). Tacrolimus is known to induce hypertension. Lazelle et al. found that this effect was primarily due to direct inhibition of calcineurin in cells expressing the renal thiazide-sensitive sodium chloride cotransporter (NCC). Thiazide diuretics may be particularly effective in improving tacrolimus-induced hypertension (Rebecca et al., 2015). Moreover, the RhoA/ROCK pathway, like the one mentioned above, enhances Ang II-specific vasoconstriction, reduces GFR and decreases proteinuria, which also contributes to secondary hypertension. However, this process has been shown to be blocked by the RhoA/ROCK inhibitor fasudil (Xiaohua et al., 2022). The latest research suggests that a newly discovered calcium channel subunit, α2δ-1, leads to CNI-induced increases in synaptic N-methyl-D- aspartate receptors (NMDAR) activity in presynaptic sympathetic neurons of paraventricular nucleus (PVN) of the hypothalamus and sympathetic outflow, which leads to hypertension. Gabapentinoids (gabapentin and pregabalin) can be reemployed for the treatment of CNI-induced neurogenic hypertension (Jing-Jing et al., 2023). In addition, infections, gastrointestinal disorders, hyperkalemia, dyslipidemia, and other adverse reactions associated with tacrolimus are also worth noting.

## 5 Efficacy and tolerable safety of tacrolimus in the treatment of lupus nephritis

The efficacy of tacrolimus in treating LN has been validated by numerous clinical studies. A meta-analysis showed that tacrolimus or multi-target (TAC + MMF) was more effective than intravenous cyclophosphamide in inducing renal complete remission in moderately severe lupus nephritis, and tacrolimus may be more effective in reducing proteinuria, with potential effects on long-term outcomes (Jennifer et al., 2015). Similarly, a study by Li et al. illustrated that tacrolimus combined with MMF was more effective than MMF alone in LN treatment; however, the former may entail a higher risk of serious adverse effects than monotherapy (Young Ho and Gwan Gyu, 2021). Furthermore, tacrolimus demonstrated superior efficacy, particularly in inducing remission in active LN (grades III/IV/V) or refractory LN (grades IV/V), as demonstrated by adaptive testing conducted in Asian populations (Andreas et al., 2019). A randomized controlled trial with long-term follow-up showed that after 60.8 ± 26 months of treatment, 24% of patients in the MMF group developed proteinuria, 18% had a flare-up of nephritis, and 21% developed renal function abnormalities (≥30% decrease in creatinine clearance or progression of chronic kidney disease to stage 4/5 or death) compared with the probability of the above in the tacrolimus group, which was 35%, 27%, and 22%, respectively (Chi Chiu et al., 2015). Although the long-term use of tacrolimus poses toxicity concerns, it also demonstrates acceptable safety under significant clinical efficacy. In LN, a study with a mean treatment duration of 309 days showed a relatively low overall incidence of adverse events (AEs) at 20.8%, with adverse drug reactions (ADRs) in 9.8% of patients. Of these, most events (69.4%) were mild, and only 37.8% of all adverse events were deemed possibly related to tacrolimus. Tanaka et al.'s study was a prospective study with a population of young adults, a mean treatment duration of 42 months and an induction maintenance dose of tacrolimus of 3 mg/d. The results showed that long-term, low-dose immunosuppressive therapy based on tacrolimus was beneficial and had low cytotoxicity, which proved that tacrolimus was an attractive choice for young patients with LN (Hiroshi et al., 2013). More interestingly, some studies have shown that tacrolimus has no negative impact on fertility of young women, and it is safe during pregnancy and lactation, which is superior to MMF and CYC (Carina et al., 2016).

In addition, the efficacy and safety of tacrolimus in the treatment of LN vary greatly among individuals, which may be related to some genetic variants affecting the pharmacokinetics of tacrolimus. The most studied enzyme system is the cytochrome P450 3A (CYP3A) family enzyme system, especially its two major congeners, CYP3A4 and CYP3A5. More studies have focused on patients undergoing kidney transplantation. The association between CYP3A5 genotypes and tacrolimus pharmacokinetics is well established (Dennis et al., 2013). Miura et al. suggested that the effect of CYP3A4 polymorphism on tacrolimus pharmacokinetics was approximately twofold smaller than that of CYP3A5 polymorphism in renal transplantation patients (Masatomo et al., 2011). Muraki et al. showed that tacrolimus pharmacokinetics in patients with autoimmune disorders were similarly affected by CYP3A5 as in transplant patients. At similar doses, CYP3A5 genotypes can significantly affect tacrolimus clearance in patients with LN. Patients expressing CYP3A5 genotypes had significantly lower tacrolimus concentrations and concentration/dose ratios and a significantly higher incidence of adverse reactions (Yuichi et al., 2018). The interaction between CYP3A4/5 polymorphisms and tacrolimus efficacy is incompletely understood, and more comprehensive studies are needed to continue to explore this in the future.

## 6 Conclusion and future recommendations

This article focuses on the role of tacrolimus, a calcineurin phosphatase inhibitor, in the treatment of lupus nephritis. Firstly, various factors leading to systemic lupus erythematosus and lupus nephritis are elucidated. Subsequently, we delve into the mechanism of action of tacrolimus. In terms of immune mechanisms, tacrolimus inhibits multiple key signaling pathways, including the regulation of NF-AT transcription factor, suppression of the expression of key cytokine genes such as IL-2, TNF-α, IL-1β, downregulation of CD40 ligand leading to the weakening of the interaction between T cells and antigen-presenting cells and B cells, thereby alleviating systemic inflammation. Additionally, tacrolimus also exerts effects in non-immune mechanisms, including stabilizing the actin cytoskeleton of glomerular podocytes, regulating Ang II-specific vasoconstriction to reduce proteinuria, and inhibiting the activity of P-glycoprotein. Current research results demonstrate the efficacy and safety of tacrolimus in the treatment of lupus nephritis. Especially for refractory and recurrent LN, its efficacy surpasses that of other drugs and is more favorable for young patients and pregnant women. However, clinicians need to closely monitor the potential adverse reactions of tacrolimus when administering it. It is recommended to regularly monitor the renal function, blood glucose, and blood pressure levels of patients, pay attention to signs of neurological abnormalities and infection, in order to minimize the toxicity of the drug within the therapeutic range. In conclusion, tacrolimus is an effective and tolerable drug for the treatment of lupus nephritis, but further prospective studies covering a wider range of ages and regions are needed to validate its long-term safety and applicability.
